# Validity of accelerometry in step detection and gait speed measurement in orthogeriatric patients

**DOI:** 10.1371/journal.pone.0221732

**Published:** 2019-08-30

**Authors:** Alexander M. Keppler, Timur Nuritidinow, Arne Mueller, Holger Hoefling, Matthias Schieker, Ieuan Clay, Wolfgang Böcker, Julian Fürmetz

**Affiliations:** 1 Department for General, Trauma and Reconstructive Surgery, University Hospital, LMU Munich, Munich, Germany; 2 Translational Medicine, Novartis Institute for Biomedical Research, Basel, Switzerland; Geffen School of Medicine at UCLA, UNITED STATES

## Abstract

**Background:**

Mobile accelerometry is a powerful and promising option to capture long-term changes in gait in both clinical and real-world scenarios. Increasingly, gait parameters have demonstrated their value as clinical outcome parameters, but validation of these parameters in elderly patients is still limited.

**Objective:**

The aim of this study was to implement a validation framework appropriate for elderly patients and representative of real-world settings, and to use this framework to test and improve algorithms for mobile accelerometry data in an orthogeriatric population.

**Methods:**

Twenty elderly subjects wearing a 3D-accelerometer completed a parcours imitating a real-world scenario. High-definition video and mobile reference speed capture served to validate different algorithms.

**Results:**

Particularly at slow gait speeds, relevant improvements in accuracy have been achieved. Compared to the reference the deviation was less than 1% in step detection and less than 0.05 m/s in gait speed measurements, even for slow walking subjects (< 0.8 m/s).

**Conclusion:**

With the described setup, algorithms for step and gait speed detection have successfully been validated in an elderly population and demonstrated to have improved performance versus previously published algorithms. These results are promising that long-term and/or real-world measurements are possible with an acceptable accuracy even in elderly frail patients with slow gait speeds.

## Introduction

Independence, mobility and physical activity are important factors for the health of orthogeriatric patients, especially in existing osteoporosis. Treatment of orthogeriatric patients aims to achieve satisfactory mobility and pain relief with surgical or non-surgical treatment methods [1]. In the field of musculoskeletal research, physical activity is a parameter directly influenced by various treatment options and is increasingly recognised as an important outcome measure [2,3].

Gait speed is one possible parameter to describe physical functional ability of patients. It has been used in the evaluation of multiple sclerosis and other neurological diseases [4] to objectively measure physical health status, and has been shown to be prognostic of increased risk of mortality [5]. In practice, gait speed is often measured in controlled environments as part of the Short Physical Performance Battery or other clinical procedures like the 6-minute walk test [6,7]. These tests are commonly used, but they only capture specific, short periods of time and do not quantify the usual, real-world gait speed [8]. Partially, also standardized questionnaires are used to conclude about the mobility of patients. Multiple studies showed that there is no correlation between a questionnaire and the measurement of physical activity in patients. Patients significantly overestimated their daily activity and walking time [9,10]. But only with reliable information about real-life walking behaviour, conclusions can be drawn about how different treatments influence the mobility of patients and whether mobility parameters are relevant for the long-term outcome of patients.

Elderly people or patients with slow or impaired real-world gait speed, typical orthogeriatric patients, have not been addressed so far, despite the relevance of gait speed to management and treatment of those patients. In this group of patients, the gait pattern is often more difficult to record and associated with high technical requirements due to the physiological peculiarities of ageing. Due to this, the accuracy of the detection is limited, especially with slow gait speed [11].

Accelerometry is a frequent component of modern portable devices and various activity parameters can be calculated with the help of specific algorithms. High-resolution accelerometry (100 Hz) is a simple and robust technique, and can provide parameters like step count, activity count or caloric expenditure [12]. The assessment of gait speed from accelerometry data is technically challenging and requires validation in the intended patient population, for example Multiple Sclerosis [13]. In addition, a reliable recording of step count is also possible, which is a generally understandable measure of the physical activity [14].

The aim of this study is to improve and validate continuous measurements of mobility in an orthogeriatric population using accelerometry data for real-world gait speed estimation. Simultaneous assessment of reference speed in a real-life walking parcours, which simulates real-world situations, is used to improve performance of step detection and real-world gait speed estimation algorithms for application in capturing real-world gait speed of elderly, slow-walking patients.

## Methods

The study was approved by the local ethical committee (Ref. 627–16) and written consent was obtained by every subject.

For this study, 20 elderly subjects without walking aids or current gait impairment due to neurological disease or acute injury, were recruited from an orthogeriatric population suffering osteoporosis.

The subjects were asked to walk along a gait course (“parcours”) which was specifically designed to include real-world environments and scenarios and included both indoor and outdoor sections. The goal was to replicate everyday life as closely as possible, the parcours therefore excludes the motivational and other psychological aspects that are usually used during gait tests in a fully controlled clinical environment.

Subjects started their walk in a hallway of the hospital, at the end of which they reached a staircase with 23 steps descending to reach the lobby of the clinic. Crossing the lobby, the subjects went outdoors, where they walked down a pathway towards a road. On their way to the road, subjects walked down a ramp to reach the pavement and ascended 5 steps to reach the pathway back to the clinic. From here on the subjects turned and walked the same path back to the starting point.

While performing the parcours, the subjects wore an actibelt^®^; a tri-axial accelerometer with a sampling frequency of 100 Hz, placed inside the belt buckle, and fixed around the waist by either a leather or elasticated belt, in order to be both discreet and located close to the subject’s centre of mass.

Reference standard data for the distance walked and the real-world gait speed during the parcours was collected using a “perambulator”: an actibelt® mounted on a calibrated measurement wheel (M10, Geofennel, Baunatal, Germany) to enable the development and cross-validation of algorithms for the estimation of real-world gait speed, based on the accelerometry data, and operated by an observer following the subject. Based on these data, precise mean values for the real-world gait speed of the subjects can be derived from the sinusoidal acceleration signals of the accelerometer placed near the rotational axis of the wheel for up to every 25 cm of the parcours. This can be considered a sufficiently accurate measure, since the step length of a person usually exceeds a length of 25 cm [15].

To assess the quality of step detection algorithms and their derived output for gait characteristics, such as the step, stance or swing time and the step count, the subject‘s walk was documented on video with a high-definition smartphone (Huawei, Mate 9, Shenzhen, China). Reference data using video annotation is well established for validation of accelerometry data since many years [16]. We have therefore opted for this method, which allows several possibilities in the post-processing.

Statistical analysis was performed with SPSS Version 24 and R version 3.3.3.

## Results

19 of the 20 subjects recruited, were female, which is not unexpected as osteoporosis is more frequently observed in women. The demographic data of the population is shown in Table 1.

**Table 1:** Orthogeriatric study population.

Demographics	Median (SD)
N	20
Age	75.5 (7.817)
Height (cm)	162.5 (7.409)
Weight (kg)	67 (11.119)
BMI	26.175 (3.575)

Different comorbidities are also to be expected within the given patient group, Table 2 displays a summary of comorbidities and indicators observed for the subjects of the study.

**Table 2:** List of comorbidities.

Comorbidity	Proportion (%)
Hip fracture	20
Vertebral body fracture	20
Wrist fracture	35
Cardiovascular disease	45
Neurological disease	20
Musculoskeletal disease	70
Previous falls	30
Joint arthroplasty	25
Sports on a regular base	55

### Range of speed

**Fig 1:**
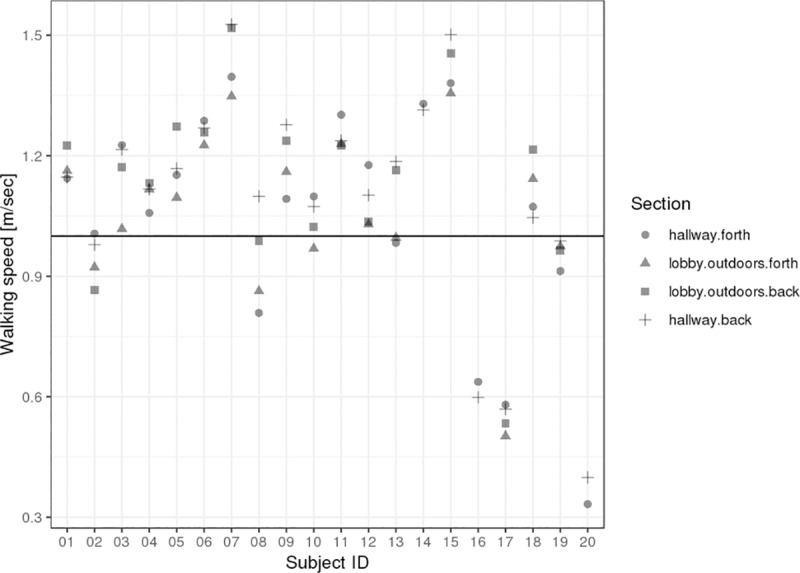
Range of speed. Variability of the mean reference speed per parcours section as measured with the measurement wheel for each subject.

Six out of 20 subjects were in the gait speed range below 1.0 m/s. Most subjects (n = 14) are in the range between 0.9 m/s and 1.2 m/s. Mean speed values for each segment of the parcours based on the reference measurements are shown in Fig 1.

For subject 16 and 20 the parcours had to be shortened to a simpler back and forth walk in the hallway section, thus data is only shown for the completed sections of the parcours.

### Algorithms

We tested two sets of algorithms for step detection and speed estimation. As a reference we used the original algorithms developed for use with actibelt^®^ and validated in healthy volunteers [17] and MS patients [13] and we tested a new set of algorithms designed to improve step detection and speed estimation in elderly, slow-walking adults.

The accelerometer is placed in a specific position inside the belt buckle. Therefore if the belt is correctly worn one of the axis of the accelerometer approximately corresponds to the direction of gravity. We therefore use the reference frame of the accelerometer as our algorithm does not correct the direction of gravity.

The original algorithms consist of the algorithm for step detection, named *stepslc*, and for speed estimation, named *speedsvr*. The step detection algorithm *stepslc* applies a sliding window approach with adaptive thresholds for magnitude and frequency to detect minima in the vertical acceleration signal, which are assumed to correspond to heel strike events. Detected steps are subsequently used to estimate a mean speed per step using *speedsvr*, a support vector regression machine trained normal walking volunteers to estimate the walking speed based on 4 different features extracted from the accelerometer signal [18].

The newly developed algorithm, named *stepwave*, builds on the feature extraction approach for step detection and speed estimation. Individual raw acceleration data files for a given patient were processed using an algorithm similar to Sabatini et al where first steps are detected and parameterized, before a Hilbert transform is used to calculate an analytical signal from which gait speed is projected per step using a linear model [19]. In the first step, a short time Fourier Transform is used to extract dominant frequencies from the raw signal: a broad band (0.7 Hz– 3 Hz) filter pass removes some noise from the signal before it is divided into overlapping windows of approximately 2.5 seconds, a Fast Fourier transform (FFT) then calculates the frequency domain for each axis. For each window, these results are then combined to determine the dominant frequencies, removing windows where the angle toward gravity or overall activity is not plausible for upright walking, or where there is no dominant frequency. For windows that pass these checks, a Butterworth filter is applied and a Hilbert transform is used to determine the frequency (F), phase (P) and amplitude (A) for each axis (vertical, x; lateral, y; and longitudinal, z). Ax, Ay, Az give good indication of the force involved in a step, independent of the exact timepoint, while F indicates step frequency, and P yields the relative position within a step. Finally, to predict gait speed, a linear model is fit to the parameters Ax, Ay and Az and their interaction terms from our training set (see below). Those features can subsequently be used to detect steps in the signal and estimate mean speeds per step. For the speed estimation a linear model has been fitted to the extracted features using a different dataset (see validation dataset [18]).

### Comparison of step counts

**Fig 2:**
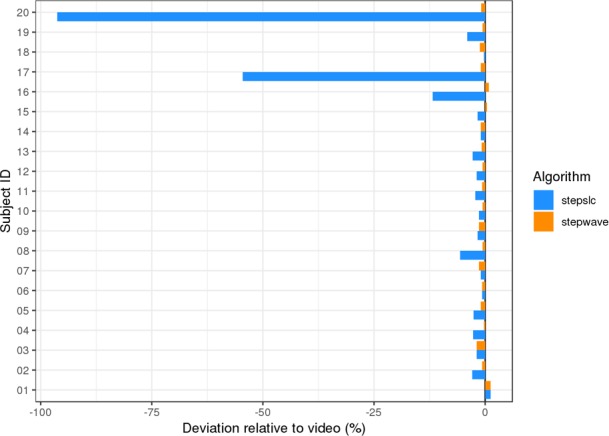
Comparison of step counts. Comparison of the deviation from the video recorded steps (estimated steps divided by video steps minus 1) in percent for the two step detection algorithms. The vertical line at 0 depict identical step counts for video and algorithm.

Annotations for gait events such as the heel strikes, the toe offs and the overall step count was extracted from video data captured during each parcours and used to cross-validate step detection algorithms. Since the current implementations of the step detection algorithms do not yet yield toe-off events as an output, to compare the methods within the context of this paper only the overall step count is used. During the video documentation one of the subjects (subject 14) could not be recorded throughout the outdoors sections of the parcours due to a technical issue, and hence these sections were removed from this subject from the comparison displayed in Fig 2.

Based on Figs 1 and 2, the original *stepslc* algorithm shows reduced step detection performance for gait speeds below 0.8 m/s (in particular subjects 16, 17 and 20).Relative to this, the *stepwave* algorithm demonstrates good performance in the lower speed range and a more consistent performance over the different subjects and gait speeds.

### Comparison of gait speed estimation

**Fig 3:**
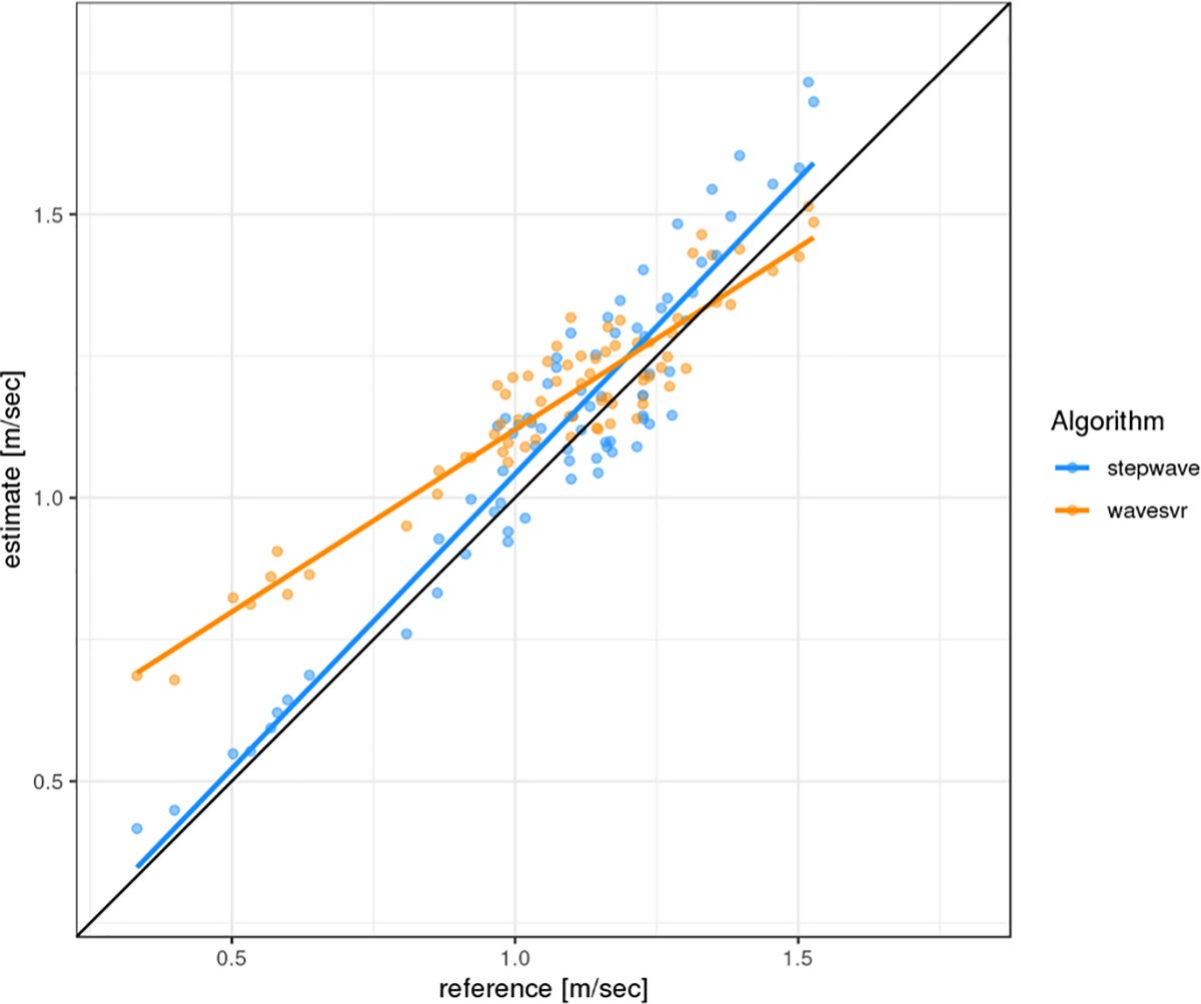
Scatter plot of mean speeds for each section of the parcours. Displayed are the speed estimates of the two algorithm combinations with identical step detection plotted against the corresponding reference measures. The algorithms use the accelerometry signal from the belt mounted actibelt®.

The original speed estimation algorithm combines the step detection by *stepslc* with the speed estimation performed by *speedsvr* for the detected steps. However, for the comparison of speed estimates we combined step detection from *stepwave* with *speedsvr* to eliminate the discrepancies in *stepslc* step detection. We call this combination of algorithms *wavesvr*. Fig 3 displays the estimates of the original speed algorithm in comparison to the reference speeds as captured with the measurement wheel. Since *speedsvr* has been trained with data from a healthy population walking in a normal speed range, the original speed algorithm is expected to perform reasonably well in a similar speed range [17]. However, this combination is known to overestimate speeds in the lower range (<0.8 m/sec) [13], hence the increasing deviation from the reference (black diagonal in Fig 3) with decreasing gait speed. Thus, the development of the new *stepwave* algorithm has been especially focused on the lower speed range.

The stepwave algorithm consists of step detection and speed estimation, but only the speed estimation part requires training. For the training of speed estimation we used the original dataset published by Schimpl et. al. [18].

While *stepwave* was trained on a similar dataset as *speedsvr* with healthy subjects and includes some artificially slow walks, speed estimates are close to the reference speed even below 0.5 m/sec of observed gait speed in the independent validation data (Fig 3). The linear fit for stepwave estimates is much closer to the diagonal (intercept of 0.002 and slope 1.040) compared to *wavesvr* (intercept of 0.477 and slope of 0.643) indicating an overall performance improvement in *stepwave*.

Overall *stepwave* shows less bias over the whole range of speeds and in particular in the speed range below 1.0 m/s. A comparison of the mean deviations for a given speed range is shown in Table 3, the difference of *wavesvr* is about five times higher than for *stepwave* for speed < 1.0 m/sec (t-test of the difference with from the mean of 0 p < 0.0001) whereas for speed ≥ 1.0 m/sec the difference is about the same (p > 0.9). This shows that *stepwave* significantly improves estimation of low gait speed, for higher gait speed the two algorithms do not perform significantly differently in our sample.

**Table 3:** Mean difference between reference speed and estimated, grouped by speed range. Both listed speed estimation algorithms utilize the stepwave step detection approach.

Speed range	Mean reference speed (m/s)	Mean estimated speed (m/s)	Difference (m/s)	Algorithm
Reference speed < 1 m/s	0.779	0.985	-0.206	*wavesvr*
1 m/s ≤ Reference speed < 1.5 m/s	1.200	1.250	-0.045	*wavesvr*
Reference speed < 1 m/s	0.779	0.818	-0.039	*stepwave*
1 m/s ≤ Reference speed < 1.5 m/s	1.200	1.250	-0.048	*stepwave*

**Fig 4:**
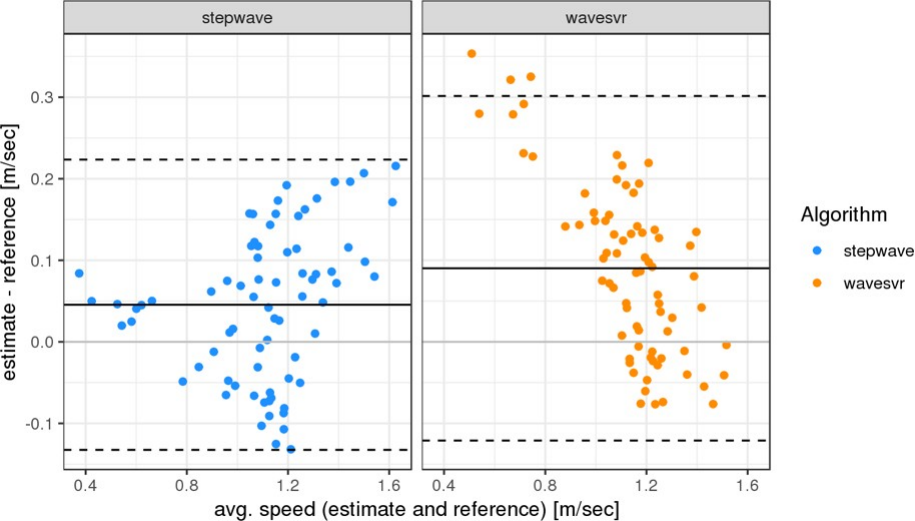
Bland-Altman plots of the applied algorithm combinations. The plot for the original speed algorithm wavesvr and stepwave. The solid horizontal lines are the mean differences between estimated and reference speed, and the dashed lines the 1.96 fold standard deviations.

The Bland-Altman plot in Fig 4 indicates an overall increased accuracy between the reference and *stepwave* estimates, since the errors mostly agglomerate within the limits of agreement or barely lie beyond, as opposed to the *wavesvr* estimates for lower speed.

## Discussion

This study aimed at implementation of a validation framework appropriate for elderly patients and representative of real-world settings, and to use this framework to test and improve algorithms for mobile accelerometry data in an orthogeriatric population. We present a study setup for the collection of high resolution accelerometry and reference data from elderly patients under realistic everyday walking conditions. We then describe how these data can be used to improve of speed estimation and step detection in slow walking orthogeriatric patients.

In previous studies, the original algorithms *stepslc* and *speedsvr* showed a speed overestimation, e.g. in multiple sclerosis patients with moderate to severe disability [13]. With the new algorithm *stepwave*, step detection notably improved for slow walkers (for subjects 16, 17 and 20 with gait speed < 0.8 m/s) compared to the original *stepslc* algorithm (see Figs 1 and 2). The accuracy of speed estimation was significantly higher than with the original algorithm *speedsvr* (Figs 3 and 4). Gait speed estimation improved for subjects with slow gait speeds (see Table 3. The linear regression figure and Bland-Altman plots (Figs 3 and 4), highlight the improvement in speed estimation.

Recently published algorithms for speed estimation, using accelerometry on a treadmill providing the ground-truth speed values, showed lower accuracy (RMSE 0.11–0.16 m/s vs. 0.09 m/s in our study) [20]. To our knowledge, there is no other algorithm published which transforms accelerometry data from an elderly population into gait speed with comparable accuracy in ecologically valid settings.

In contrast to gait speed, for which there are few published studies, step detection is well studied with multiple devices on multiple populations [21–26], however different validation protocols, populations and test scenarios make a comparison between existing results challenging, and there is no consensus about standardized setting [21,27,28]. Khan et al. and Lipperts et al. tested and validated their algorithms in an elderly population[27,28]. Lipperts et al. used a skin-mounted device in a healthy population and compared it to an orthopaedic target population in a real-life scenario, reporting accuracy in step detection above 92%[27]. Khan et al. tested their algorithm on healthy elderly home-living persons without abnormal gait patterns and showed accuracy of 94.4%[28]. However, impaired gaits with asymmetry and slow gait speeds downgrade the accuracy of algorithms detecting steps significantly [23]. In contrast to this, the presented *stepwave* algorithm is still highly accurate for step detection in elderly people including slow gait speeds (see Fig 2). In order to generate evidence on various surgical and non-surgical treatment options, objective, longitudinal measurements of a patient’s physical function are needed. Wearable physical activity monitors (PAMs) can now deliver long-term information about the activity of the individual [29]. This is a new opportunity to constantly monitor patient’s outcome over a certain time period and collect data on daily activity patterns and real-world behaviour [30]. Increasingly, these devices are finding their way into medical applications [31].

The presented assessment protocol in our study includes a real-world walking scenario and thus stands out clearly from other scenarios, e.g. testing on a treadmill or in the gait laboratory which can deviate strongly from walking representative of real-world gait speed [23,32]. The difficulty is to measure the physical activity under real-life conditions in a highly accurate manner.

A clear limitation of our study is that the number of patients included is low. As a result, few people are available for validation of slow gait speeds. Whether the described *stepwave* algorithm achieves a similarly high accuracy in a larger number of slow walking patients or in patients with even severe more gait impairment and very slow gait speeds is the subject of further studies. Furthermore, we excluded the stairs from the speed calculation. Here measurement with a rolling perambulator wheel is not working, so for this part of the parcours only the step detection is included.

## Conclusion

The applied study setup and reference measurements have proven to deliver reliable and high-quality data, which can be fully used to improve algorithms to extract objective measures from accelerometry data.

Further analysis including an analysis of gait characteristics using annotated video data, as well as a comparison of the performance of various step detection and real-world gait speed estimation algorithms is warranted. Future research, for example in orthogeriatric and frail patient populations are necessary to achieve more detail and data especially for slow walking speeds and impaired gaits.

By improving accuracy of mobility assessment with accelerometer devices we can achieve a magnitude of insight information about long-term mobility in different populations including frail and elderly patients.
