# Correction: Validity of accelerometry in step detection and gait speed measurement in orthogeriatric patients

**DOI:** 10.1371/journal.pone.0337268

**Published:** 2025-11-24

**Authors:** Alexander M. Keppler, Timur Nuritidinow, Arne Mueller, Holger Hoefling, Matthias Schieker, Ieuan Clay, Wolfgang Böcker, Julian Fürmetz

After publication of this article [1], it was raised that there is a missing affiliation for author Timur Nuritidinow. The correct affiliations for Timur Nuritidinow are Sylvia Lawry Center of MS Research e.V. - The Human Motion Institute, München, Germany and the Department for General, Trauma and Reconstructive Surgery, University Hospital, LMU Munich, Munich, Germany.

The Funding and Competing Interests statements have therefore been updated to the following:

Funding: The Sylvia Lawry Center for MS Research e.V./(SCLMSR), a registered non-profit association in Munich, provided partial funding for the costs of the ethics vote and the insurance. Arne Mueller, Holger Hoefling, Matthias Schieker and Ieuan Clay are employed by Novartis Institute for Biomedical Research. Novartis Institute for Biomedical Research provided support in the form of salaries for authors MS, IC, HH, and AM, but did not have any additional role in the study design, data collection and analysis, decision to publish, or preparation of the manuscript. Timur Nuritidinow was employed by SCLMSR e.V. during data collection. The specific roles of these authors are articulated in the author contributions section.

Competing interests: We have the following interests: Arne Mueller, Holger Hoefling, Matthias Schieker and Ieuan Clay are employees of and may hold stock in Novartis Institute for Biomedical Research and Novartis Pharma AG. Matthias Schieker is a Professor and member of the medical faculty at the University of Munich (LMU). Timur Nuritidinow was employed by the Sylvia Lawry Center for MS Research e.V. (SCLMSR) during data collection. Martin Daumer and Christian Lederer from Trium Analysis Online GmbH and SCLMSR e.V. in Munich provided the actibelt® and technical support. There are no further patents, products in development or marketed products to declare. This does not alter our adherence to all the *PLOS One* policies on sharing data and materials.

There are also errors in the Author Contributions. The correct Author Contributions are:

**Conceptualization:** Alexander M. Keppler, Holger Hoefling, Matthias Schieker, Ieuan Clay, Julian Fürmetz.

**Data curation:** Timur Nuritidinow, Arne Mueller.

**Formal analysis:** Timur Nuritidinow, Arne Mueller, Holger Hoefling, Ieuan Clay.

**Investigation:** Alexander M. Keppler.

**Methodology:** Julian Fürmetz.

**Project administration:** Alexander M. Keppler, Matthias Schieker, Ieuan Clay, Wolfgang Böcker, Julian Fürmetz.

**Resources:** Wolfgang Böcker.

**Software:** Timur Nuritidinow, Arne Mueller, Holger Hoefling.

**Visualization:** Arne Mueller, Holger Hoefling.

**Writing – original draft:** Alexander M. Keppler, Timur Nuritidinow, Julian Fürmetz.

**Writing – review & editing:** Arne Mueller, Holger Hoefling, Matthias Schieker, Ieuan Clay, Wolfgang Böcker, Julian Fürmetz.
